# Emerging stock market reactions to shocks during various crisis periods

**DOI:** 10.1371/journal.pone.0272450

**Published:** 2022-09-13

**Authors:** Roni Bhowmik, Gouranga Chandra Debnath, Nitai Chandra Debnath, Shouyang Wang

**Affiliations:** 1 School of Business, Guangdong University of Foreign Studies, Guangzhou, China; 2 Department of Business Administration, Daffodil International University, Dhaka, Bangladesh; 3 School of Business & Economics, United International University, Dhaka, Bangladesh; 4 Kalinga Institute of Industrial Technology (KIIT), Bhubaneswar, India; 5 Bangladesh Academy for Securities Markets, Dhaka, Bangladesh; 6 Academy of Mathematics and Systems Science, Chinese Academy of Sciences, Beijing, China; German University in Cairo, CZECH REPUBLIC

## Abstract

This study investigates granger causal linkages among six Asian emerging stock markets and the US market over the period 2002–2020, taking into account several crisis periods. The pairwise Granger causality tests for investigating the short-run causality show significant bi- and uni-directional causal relationships in those markets and evidence that they have become more internationally integrated after every crisis period. An exception is Bangladesh with almost no significant short-term causal linkages with other markets. For understanding, how the financial linkages amplify volatility spillover effects, we apply the GARCH-M model and find that volatility and return spillovers act very inversely over time. However, market interface is weak before the crisis periods and becomes very strong during the financial crisis and US-China economic policy uncertainty periods. The US market plays a dominant role during the financial crisis and COVID-19 periods. Further analysis using the VAR model shows that a large proportion of the forecast variance of the Asian emerging stock markets is affected by the S&P 500 and that market shock starts to rise notably from the 1 to 10 period. The overall findings could provide important policy implications in the six countries under study regarding hedging, trading strategies, and financial market regulation.

## 1. Introduction

From the time of 1980s degree of causal linkages of financial and stock markets around the world increased significantly [1]. This correlation and connectivity in the financial markets are worldwide increasing because of globalization and financial liberalization and the increasing size of cross-border trades as well as financial flows. This is why, investors, financial institutions, and governments are very interested in understanding the correlation and effect of the relationships among different financial markets. Moreover, international portfolio investors are concerned with the emerging markets because they can be a prevalent destination to obtain diversification investment benefits. Over the past few decades, there has been increasing importance in the role of the Asian financial markets and getting more attention from global investors. According to several studies, the global financial crisis 2007–2008 and US-China trade despite 2018–2019, and the COVID-19 pandemic has a great effect on the stock markets [2–4]. Thus, it is interesting to examine the Granger causal linkages among the Asian emerging stock markets and the US stock market in recent crisis periods.

First, the global financial crisis in 2007–2008 tremendously affected the world economy. The global financial crisis was started by subprime mortgages and the academic researchers mentioned that this was manifested in the increase in the correlation and interactions in global financial markets [5–7]. Second, the stock markets of China have been adversely affected by the US-China trade dispute and this has affected their neighboring emerging countries [8, 9]. Third, the latest challenge for the economy has been the COVID-19 pandemic which has dramatically affected the demand and supply in the global economy as well as investments. Unfortunately, COVID-19 not only has impacted the production process but impacted the financial markets. The stock market volatility reached levels unseen since the global financial crisis of 2007–2008. The global stock market reacts very harshly compared to other previous crises like the Great Depression (October 1929), Black Monday (October 1987), and the global financial crisis (2007–2008) [10]. The impact of the COVID-19 is a common notion that harms the global stock markets [11–15]. Those financial crises have a negative effect on the market volatility pattern of stock market returns which differs from one country to another country according to their economic and stock market structures [6, 16].

Stock market investment decisions are directly and indirectly affected by a market causal linkages and volatility spillover effect, which has significant value for market investors and policymakers. Recently “spillover effect” is a very popular word for the financial market researchers. It has special meaning in the financial world as, co-movement, contagion, and cointegration are more common. Generally, in the crisis period cross-countries transmission happened and it is greater during this period but the spillover effect increases in the post-crisis period [7, 11]. Emerging stock market have played an important part in outfitting to the influence of different financial crisis period [7, 16]. As, those six Asian emerging stock markets is consistent with major financial markets of the world. So, it’s very important to dividing the full time period as several sub crisis periods for better understanding of the impact of the different crisis periods on the changing patterns in the cointegration and volatility spillover among the six Asian emerging stock markets (Bangladesh, China, India, Malaysia, Philippines, South Korea) and the US market. The motive for choosing these emerging economies stock market is that these countries’ economies are totally interconnected with main financial markets in the world. There can be a higher possibility of spillover effects of the different crisis periods on these Asian emerging economies.

An extensive literature is existing on the spillover effect of the different crisis on the emerging stock markets. Lee et al. (2004) studied the linkages between the US and Asian stock markets and found a solid indication of volatility spillovers from the US to Asia [17]. They have mainly focused on the more developed markets which are highly interconnected with each other and the volatility of the US market is spread to other developed markets more quickly. However, in the present world, more than 60% of global GDP, arises from emerging and developing economies but the parts of this originated in the past decade [18]. Unquestionably, the previous two eras’ roles of emerging markets have developed more significantly and led to more inter-market relationships. For this reason; investors, economists, and researchers are giving extra care to the emerging stock markets [7, 8, 13, 19–24]. Yet, it remains relatively unclear whether, and to what extent, the recent crises spillover affected the Asian emerging stock market and how these markets are cointegrated with the US markets for the long term or short term and what is the current cointegration level of these markets. This paper tries to answer these questions and adds contribution to those crises’ periods literature by examining the emerging Asian markets through an analysis of Granger causality linkages and market integrations, as well as return and volatility spillovers with respect to the US stock markets during various crisis periods.

The core contribution of this paper is established in the following three facets: (1) the sample period which covers data over the period 2002 to 2020, which has not been considered in an earlier investigation of Asian emerging markets. (2) The examination of various crisis periods to better understand at what period the markets are flattering more causality and volatility and influenced by external shocks from US stock markets. (3) The application of several econometric models, namely the Granger causality tests for analysis of the short-run causality relationships among those markets; the Generalized Auto-Regressive Conditional Heteroskedasticity in mean (GARCH-M), and Vector autoregression (VAR) model to capture how much possible market volatility news affects the dynamic connections with the US stock market.

## 2. Literature review

The application of causality tests is not a new idea for financial market analysis and financial market researchers [25, 26]. A large number of studies investigate the causal linkages in the stock markets between both developed and emerging markets. Most of the studies considered developed markets, especially the US and evidence provides that the US stock market latter leads other developed stock markets [6, 14, 15, 27–31]. And many previous studies have investigated the relationship between the stock market in various crisis periods e.g. Great Depression, Black Monday, Asian financial crisis, global financial crisis, European sovereign debt crisis, the US-China economic policy uncertainty (EPU) (*trade war period between the US-China and in this paper we used the focused name similar as other researchers “US-China economic policy uncertainty (EPU)” period [8, 9]*), and recent COVID-19 pandemic. Though fewer studies exist on stock market causal linkages between developed stock markets and emerging stock markets [32–37]. This paper is getting inspiration from those pieces of literature to shed some further light on the issue of possible causality between developed US stock markets and Asian emerging stock markets in various crisis periods. Additionally, we contribute to the literature by expounding on the effect of the global financial crisis, US-China EPU, and the recent COVID-19 pandemic on the Asian emerging stock markets.

Firstly, there are many scholars investigating the relationship between the global financial crisis and the stock markets of different countries. Since the crisis was caused by the subprime mortgage, several studies have investigated the relationship between the global financial crisis and Asian stock markets [7, 38–43]. Most of the researchers found that the global financial crisis period has a strong influence on the Asian market and during this period the US impact increased on Asian stock markets. Secondly, the US-China EPU crisis. As the US and China are the most powerful economy in the world, in consequence, the US-China EPU crisis not only weakened those two countries’ trade relationships, they also influenced the global stock markets. Since the crisis was caused by the trade war, several studies have examined the relationship between the US-China EPU and Asian stock markets [8, 9, 44]. According to their findings market interface becomes strong and the Chinese market has more impact on the Asian stock markets during the crisis period. Finally, the COVID-19 pandemic crisis. This crisis differs from the previous two crises and it was caused by health problems. Many studies examined the impact of the COVID-19 pandemic on emerging stock markets [11, 13, 14]. Researchers mentioned that the COVID-19 pandemic crisis is the worst crisis since the Great Depression.

The previous studies have found evidence that international stock markets have significant causal linkages [24, 38, 39, 45–47] while others have found weak causal linkages [48–51]. The empirical data on stock market interrelationship is mostly assumed through the Granger causality test [52]. In the emerging markets, financial liberalization leads to the efficiency hypothesis, and financial liberalization Granger causes stock market efficiency [47]. Through causality testing, it is found that a bi-directional causal relationship exists between Indonesia and Thailand markets [53]. In South Asian countries Bangladesh, India, and Sri Lanka Granger cause the Pakistani stock market prices [54]. The linkages between the selected Asian and the US stock markets show no stock market is playing a very dominant role in influencing other markets, except the US [55]. However, India Granger causes some other South Asia regional markets and the effect is bi-directional [40]. In the Chinese stock market, most of the evidence indicates a one-way Granger causality between stocks [56].

Some researchers remark that the market volatility return in more developed and advanced markets is affected more by the news originating from the US stock market [57, 58]. Particularly, more opening of stock markets makes them more subject to the news arrived from the US stock market, especially bad news. Scholars agree that most of these Asian emerging markets’ investors do not only respond to native market news but also, they respect the news coming from more developed markets [59]. Liu et al., conduct a broad study on the structure of the global transmissions and mention that after the US stock market performs a leading character and that Singapore and Japanese markets jointly have an important persistent influence on the other Asian stock markets [60]. Ng (2000) examines the effects of market volatility through the GARCH model and finds that volatility is transmitted from Japan and the US markets to the Pacific-Basin region and the US stock market news impact is strong in this region [61]. Choudhry uses a GARCH-M model to study the stock market volatility and the determination of shocks to the volatility before and after the market crisis [62]. Our current paper keeps on the similar GARCH-M model but covers the study before the crisis, crisis, and after the crisis time period.

In an emerging market, stock returns volatility has tremendous room, on the opposite side, developed stock markets are more constant, and that is why market investors are more concerned and interested in emerging stock markets [63]. This work is comparable to Chiang et al., (2007) who have studied the nine selected Asian markets and found that market correlation increased in the financial crisis period which is called contagion consequence, and determined the extreme correlation in the few months of the market crisis, and this is mentioned as too much herding behavior in the markets [64]. Yilmaz (2010) shows that the global financial crisis has supported to get the situation on the volatility of stock market returns in East Asian countries [41]. From the prospect of South Asia, it is found that the financial crisis has negatively affected the stock returns and negative news produces higher market volatility influence than positive news in the Indian market [42]. Specially, this has motivated us on how and to what magnitude stock market return and volatility in emerging Asian stock markets can be influenced through external shocks from US stock markets.

## 3. Data description and summary statistics

This paper uses the daily closing prices for the six emerging Asian markets of indices for the dated of 02 January, 2002 to 30 December, 2020. Initially, the work is started with full sample periods (2002–2020), We divided the market data into four sub-periods to determine the better understanding of the market reaction on different crisis periods. This sample period is split into four sub-periods; pre-crisis (2002–2006), crisis (2007–2011), post-crisis (2012–2016), US-China economic policy uncertainty (EPU) (2017–2019), and COVID-19 (2020) periods. The market indices used to cover the following- the DSEX index for Bangladesh, SSE composite index for China, BSE 30 index for India, FBMKLCI for Malaysia, PSEi Index for the Philippines, KOSPI index for South Korea, and the S&P 500 index for US stock market is selected.

Table 1 presents some basic information about the six Asian emerging stock markets. Among those markets, the Malaysian stock market is the largest in terms of market capitalization followed by South Korea, India, and China. By contrast, Bangladesh is a relatively small market. In terms of listed companies, the Philippine market is the smallest. As can be seen from the listed trading values, the global financial crisis, and other crisis like COVID-19 is clearly visible with a drop in the crisis periods.

**Table 1:** Some selected stock market indicators.

Country	**2002**	**2003**	**2004**	**2005**	**2006**	**2007**	**2008**	**2009**	**2010**	**2011**	**2012**	**2013**	**2014**	**2015**	**2016**	**2017**	**2018**	**2019**	**2020**
**No. of listed companies**																			
BD	163	163	185	195	199	211	221	197	192	433	453	481	517	543	557	572	593	611	628
CN	1223	1285	1373	1377	1421	1530	1604	1700	2063	2342	2494	2489	2613	2827	3052	3485	3584	3777	4154
IN	5650	5644	4725	4763	4796	4887	4921	4955	5034	5112	5191	5294	5541	5835	5820	5615	5065	5215	5215
MY	857	897	955	1015	1021	983	972	952	948	932	911	900	895	892	893	894	902	919	927
PH	233	234	233	235	238	242	244	246	251	251	252	254	260	262	262	264	264	265	268
KR	1512	1558	1570	1616	1689	1755	1789	1778	1781	1799	1767	1798	1849	1948	2039	2114	2186	2262	2318
**Stock market capitalization as a % of countries GDP**																			
BD	27.21			4.75	5.36	10.97	12.79	20.42	36.10	37.08		34.11	39.60	33.57	31.80	34.51	28.24	21.29	27.69
CN		30.89	22.89	17.58	41.62	126.15	38.72	70.04	66.17	45.18	43.33	41.26	57.32	74.02	65.17	70.76	45.52	59.63	82.96
IN	33.44	50.85	58.59	76.15	95.21	161.24	66.00	101.89	105.18	68.27	76.07	68.13	82.72	82.96	76.09	96.39	84.49	79.67	98.95
MY	124.72	145.93	145.59	125.77	144.80	168.06	81.99	142.99	160.26	132.78	148.38	154.78	135.77	127.08	119.43	142.82	110.95	110.77	129.66
PH	21.95	26.63	30.12	37.05	53.15	65.94	28.74	49.02	75.50	70.48	87.55	76.55	88.02	77.93	75.24	88.41	74.43	73.06	75.46
KR	39.97	46.88	54.00	76.80	79.22	95.73	44.95	88.42	95.44	79.48	92.25	90.06	81.70	83.99	83.63	109.10	81.96	90.17	133.47
**Trading value of stock traded (US$, Trillion, *Billion*)**																			
BD			*0*.*248*	*0*.*205*	*0*.*236*	*0*.*708*	*1*.*46*	*2*.*34*	*4*.*84*	*2*.*22*	*1*.*44*	*1*.*24*	*15*.*81*	*13*.*61*	*15*.*32*	*26*.*50*	*16*.*14*	*13*.*13*	*14*.*92*
CN	*338*.*21*	*388*.*00*	*511*.*49*	*392*.*36*	1.16	6.30	3.90	7.83	8.26	6.67	5.03	7.69	11.96	39.32	18.29	17.22	13.07	18.25	31.58
IN	*196*.*91*	*282*.*78*	*377*.*04*	*473*.*72*	*637*.*01*	1.09	1.04	1.05	1.06	*740*.*66*	*637*.*44*	*570*.*99*	*761*.*43*	*802*.*19*	*808*.*34*	1.20	1.29	1.28	1.94
MY	*25*.*73*	*45*.*71*	*54*.*19*	*44*.*64*	*69*.*14*	*154*.*64*	*82*.*32*	*80*.*84*	*114*.*79*	*130*.*32*	*123*.*66*	*142*.*16*	*142*.*65*	*111*.*48*	*98*.*28*	*137*.*42*	*135*.*33*	*108*.*62*	*248*.*61*
PH	*2*.*14*	*2*.*44*	*3*.*23*	*5*.*35*	*9*.*16*	*24*.*23*	*12*.*37*	*14*.*32*	*22*.*25*	*27*.*37*	*35*.*68*	*44*.*59*	*42*.*21*	*38*.*42*	*35*.*85*	*33*.*80*	*29*.*21*	*29*.*99*	*32*.*74*
KR	*580*.*98*	*457*.*21*	*525*.*82*	1.2	1.34	1.92	1.19	1.68	1.63	1.93	1.58	1.33	1.28	1.84	1.60	2.01	2.45	1.93	5.19

**Data source:** The World Bank (https://data.worldbank.org/)

### 3.1 Basic statistical analysis

For analysis purpose, the stock indices of individually stock market are converted into index return to prevent difficulties following the algorithm expressing the modification in the logarithm concerning generating the closing price of today and yesterday (Eq 1), where X_*t*_ symbolizes t day’s rate of return, *p*_*t*_ represents today’s closing price and *p*_*t*−1_ represents yesterday’s closing price.


$$X_{t}=\log\;p_{t}-\log\;p_{t-1}$$ (1)

Table 2 gives a short summary of the descriptive values demonstrating that the daily mean for each of the six equity markets’ returns is positive. Particularly, the smallest of the emerging market Bangladesh has the maximum unconditional average, and India’s market returns on the second position. Returns of the Bangladesh stock market fluctuate between the maximum of 0.226 and minimum of -0.099. Market volatility is generally extreme in emerging markets, although it is one of the supreme in the emerging Asian markets [65]. Evaluating by standard deviation, firstly dominant the Chinese market at 0.015 and the nominal in Malaysian market at 0.007. Each of these market returns has negative skewness and positive kurtosis, which means that the stock market returns perhaps will not be ordinarily distributed. Each of the market stock returns has fat tails or leptokurtic as realized in the excess kurtosis, it denotes that the GARCH model can be used for pricing these series. Stock markets have “fat tails”, it is the trend of stock markets because of the extreme outcomes in the form of the stock market bubbles and crashes [7]. Additionally, the Jarque-Bera test does not accept the normality of the market stock returns series.

**Table 2:** Descriptive statistics.

Countries	Bangladesh	China	India	Malaysia	Philippine	Korea
Mean (%)	0.0006	0.0002	0.0006	0.0002	0.0004	0.0003
Median (%)	0.0003	0.0006	0.0008	0.0003	0.0005	0.0005
Maximum (%)	0.226	0.095	0.173	0.049	0.098	0.161
Minimum (%)	-0.099	-0.088	-0.111	-0.095	-0.123	-0.106
Standard Deviation	0.013	0.015	0.013	0.007	0.012	0.013
Skewness	1.30	-0.23	0.15	-0.71	-0.38	0.02
Kurtosis	35.01	7.79	14.57	13.97	9.58	14.07
Jarque-Bera	19062	4477	25881	23620	8485	23671
JB tests’ Prob.	0.0000	0.0000	0.0000	0.0000	0.0000	0.0000
Observations	4636	4636	4636	4636	4636	4636

**Data source:** The sample size is 4,636, from January 2, 2002 to December 30, 2020.

### 3.2 Unit root test

Table 3 demonstrates the unit root tests employed on two types of data: level data (prices) and first difference data (returns) using ADF, PP, and KPSS tests [66]. The results show that unit root tests for daily stock indices price data between the index level and first differences are near to zero at all consequence levels. The ADF and PP tests denote that the null hypothesis of the presence of a unit root in the levels of each of the six emerging Asian markets index prices series cannot be rejected. The exception is noted in the post-crisis time period in Bangladesh and South Korean markets where the null hypothesis is not accepted both at the 1% level in ADF and PP tests. The KPSS test results indicate to rejection of the null hypothesis of the stationary for each of the stock market indices and data frequencies for all the periods.

**Table 3:** Unit root test (full period).

Countries	Period	Panel 1: Levels			Panel 2: First Differences		
		ADF	PP	KPSS	ADF	PP	KPSS
Bangladesh	Full Period	-1.60	-1.52	6.37***	-17.63***	-65.01***	0.13
	Pre-Crisis	-0.79	-0.78	3.28***	-32.93***	-32.93***	0.18
	Crisis	-1.27	-1.26	3.41***	-27.25***	-34.44***	0.17
	Post-Crisis	-3.46***	-3.67***	0.66**	-14.19***	-35.70***	0.13
	EPU	-0.99	-1.00	1.42***	-23.74***	-23.68***	0.38
	COVID-19	-1.03	-1.05	3.02***	-30.15***	-33.18***	0.15
China	Full Period	-1.99	-1.99	2.49***	-30.90***	-66.38***	0.06
	Pre-Crisis	2.94	2.78	0.64**	-34.37***	-34.52***	1.03***
	Crisis	-1.15	-1.21	1.39***	-35.97***	-35.99***	0.22
	Post-Crisis	-1.56	-1.37	2.27***	-15.97***	-32.76***	0.08
	EPU	-1.59	-1.60	1.61***	-27.75***	-27.75***	0.08
	COVID-19	-1.21	-1.28	1.46***	-34.58***	-33.88***	0.18
India	Full Period	0.02	0.11	8.00***	-63.59***	-63.45***	0.09
	Pre-Crisis	1.67	1.56	3.85***	-26.37***	-33.15***	0.47**
	Crisis	-1.77	-1.71	1.18***	-33.17***	-33.11***	0.10
	Post-Crisis	-1.71	-1.72	3.65***	-34.08***	-34.04***	0.15
	EPU	-1.85	-1.84	2.92***	-25.68***	-25.66***	0.08
	COVID-19	-1.82	-1.76	1.31***	-35.293***	34.893***	0.13
Malaysia	Full Period	-1.56	-1.56	7.70***	-62.12***	-62.10***	0.17
	Pre-Crisis	0.18	0.07	3.77***	-32.88***	-33.09***	0.14
	Crisis	-0.96	-0.99	1.52***	-32.27***	-32.26***	0.16
	Post-Crisis	-2.39	-2.22	1.03***	-32.44***	-32.29***	0.22
	EPU	-1.24	-1.47	1.55***	-22.05***	-26.46***	0.28
	COVID-19	-1.02	-1.07	1.48***	-31.82***	-30.95***	0.19
Philippine	Full Period	-0.65	-0.61	8.34***	-65.84***	-65.88***	0.06
	Pre-Crisis	1.12	1.26	4.04***	-32.02***	-32.02***	0.34*
	Crisis	-0.74	-0.65	1.81***	-31.89***	-31.73***	0.21
	Post-Crisis	-2.51	-2.49	3.19***	-20.84***	-34.32***	0.25
	EPU	-2.90**	-2.79**	0.29	-28.85***	-29.12***	0.15
	COVID-19	-0.81	-0.73	1.72***	-32.62***	-31.47***	0.26
Korea	Full Period	-1.73	-1.70	7.23***	-67.35***	-67.38***	0.08
	Pre-Crisis	-0.05	0.03	3.56***	-35.57***	-35.61***	0.19
	Crisis	-1.81	-1.78	1.27***	-35.18***	-35.19***	0.08
	Post-Crisis	-4.52***	-4.53***	0.85***	-35.85***	-36.01***	0.03
	EPU	-1.49	-1.49	1.34***	-17.72***	-28.50***	0.33
	COVID-19	-1.93	-1.85	1.39***	-34.08***	-33.41***	0.13

**Data source:** Panel 1 presents the statistics of the unit root tests conducted on level data of the six Asian emerging market indices, while Panel 2 presents the statistics applied to first difference data. The sample size is 4,636, from January 2, 2002 to December 30, 2020.

*Denote statistical significance at the 10% level.

** Denote statistical significance at the 5% level.

***Denote statistical significance at the 1% level.

### 3.3 Pearson correlation

Pearson correlation is considered to find the short-run relationship between the movements of stock markets. In Table 4, the Pearson correlation is used for investigating the level of correlation among the stock market returns for each period. For this purpose, squared returns are used to denote the time-varying variances of the market returns. Section A presents the full periods’ outcomes, where the correlations in most of the markets are positive and extremely correlated in the regional markets [43], the exception is only found in the Bangladesh market. Section B presents the pre-crisis period outcomes, where the correlation in the variances is extremely small in this period and is negatively correlated with the US market. Section C presents the global financial crisis periods results, the outcomes are noticeable and massive contradictory to the pre-crisis periods. The correlation increases and the most significant outcome is that correlation increases with the US market. The motivation for high correlation is that there are different trade agreements of the bilateral and multilateral as well as financial relations between more mature countries [32]. Section D presents the post-crisis periods outcomes, where one noticeable remark is that the Section D outcomes seem similar to the outcomes of Section B.

**Table 4:** Correlations of the stock returns (%).

Countries	Bangladesh	China	India	Malaysia	Philippine	Korea	US
**A. Full Periods (2002–2020)**							
Bangladesh	100						
China	-0.22	100					
India	-0.58	6.74*	100				
Malaysia	-0.38	9.41*	7.12*	100			
Philippine	-0.24	9.41*	19.66*	12.83*	100		
Korea	-0.77	10.99*	14.45*	12.99*	16.53*	100	
US	-0.55	5.21*	7.31*	8.11*	6.14	8.66*	100
**B. Pre Crisis Period (2002–2006)**							
Bangladesh	100						
China	4.89	100					
India	-3.04	0.98	100				
Malaysia	3.65	10.38*	2.02	100			
Philippine	0.96	5.44*	4.49	2.19	100		
Korea	-0.16	-0.48	10.13*	13.29*	7.25*	100	
US	-1.46	-1.01	-1.17	-0.88	-0.49	-0.65	100
**C. Crisis Period (2007–2011)**							
Bangladesh	100						
China	-1.42	100					
India	-0.95	5.80*	100				
Malaysia	-0.47	16.46*	11.80*	100			
Philippine	-0.34	8.57*	10.97*	24.42*	100		
Korea	-1.24	14.01*	11.24*	23.26*	23.85*	100	
US	-1.52	13.78*	6.64*	14.31*	-0.84	6.64*	100
**D. Post Crisis Period (2012–2016)**							
Bangladesh	100						
China	1.66	100					
India	0.01	10.81*	100				
Malaysia	-1.79	2.00	13.81*	100			
Philippine	-1.79	15.37*	51.19*	19.04*	100		
Korea	-4.23	9.53*	25.74*	11.84*	15.72*	100	
US	-1.28	2.15	16.95*	2.57	8.05*	18.58*	100
**E. US-China EPU Period (2017–2019)**							
Bangladesh	100						
China	2.50	100					
India	18.38*	6.80*	100				
Malaysia	-2.76	8.15*	1.18	100			
Philippine	-0.54	5.74*	13.31*	11.72*	100		
Korea	4.86	30.19*	13.50*	14.04*	17.75*	100	
US	-3.57	10.28*	15.54	7.91*	9.01*	4.94	100
**F. COVID-19 Crisis Period (2020)**							
Bangladesh	100						
China	1.78	100					
India	5.21*	8.15*	100				
Malaysia	1.01	15.31*	6.71*	100			
Philippine	-0.83	13.16*	8.27*	15.72*	100		
Korea	-1.01	21.13*	19.31*	27.09*	25.03*	100	
US	-2.73	8.52*	10.03*	11.36*	4.27	5.16*	100

**Note:** *Denote statistical significance at the 5% level.

Section E presents the US-China EPU 2018–2019 period results, another remarkable observation is that the Section E results appear to be very similar to the results of Section C. Although, outcomes demonstrate that emerging Asian market correlations are increasing. Section F presents the COVID-19 pandemic period results, the outcomes are noticeable in that this section’s results seem to be parallel to the results of sections C and E. During this pandemic period, most of the country’s economic indexes downwards excluding the stock market [10]. Correlation increased between emerging Asian markets with the US market during the COVID-19 period. The correlation between the US and China shows is weak. When the shock of the COVID-19 pandemic emerged, it led immediately to financial panic that spread throughout the emerging Asian stock markets.

## 4. Methodology

### 4.1 Granger causality

In order to check the short-run relationship between different stock market indices, we estimate the granger causality relationship. For additional study in this pairwise granger causality, two-way interconnection of variables, say R and S can be examined. The subsequent bivariate regression is expended where R granger causes S and S granger causes R.


$$R_{t}={A_{0}+A_{1}R}_{i-1}{+\cdots +A_{n}R}_{i-k}{+B_{1}S}_{i-1}{+\cdots +B_{n}S}_{i-k}+\epsilon_{t}$$ (2)

$$S_{t}={A_{0}+A_{1}R}_{i-1}{+\cdots +A_{m}S}_{i-1}{+B_{1}R}_{i-k}{+\cdots +B_{m}R}_{i-k}+\omega_{t}$$ (3)

The calculated F-statistics is used to accept or reject the null hypothesis of no causality.

### 4.2 GARCH-M model

If appropriate a regression model with GARCH errors that can use the conditional standard deviation *σ*_*t*_ act one of the regression variables when the dependent variable is returned, then the stock market demands a low-risk premium for lower risk, and the reverse is as the higher risk premium for higher risk [67]. So, higher conditional variability is the reason for superior returns. The GARCH-M model extends the conditional mean equation as obeys:

$$R_{i,t}=c_{i}+\gamma_{i}k_{i,t}+\varepsilon_{i,t}$$ (4)

$$k_{i,t}=\alpha_{O}+\alpha_{1}\varepsilon_{i,t-1}^{2}+{\beta k}_{i,t-1}$$ (5)

$$\varepsilon_{i,t}/I_{t-1}∼N(0,K_{t})$$

Where as *k*_*i*,*t*_ is the conditional variance of the country *i* stock market portfolio, which respects GARCH (1,1) procedure, *I*_*t*−1_ the information set accessible at the beginning of time *t* and the conditional density function is expected to have a normal error distribution.

For examining the transmission of volatility between Asian emerging stock markets and developed markets symbolized by US market, here we usage multivariate GARCH (1,1) model to recognize the cause and level of spillovers. For approximating the conditional variance–covariance matrix we employed the BEKK model. This model accepts the conditional variances and covariances of the markets to effect respectively. Simultaneously, confirms the condition of an optimistic semi-definite conditional variance–covariance matrix in the optimization manner.


$$R_{i,t}=\alpha +{JR}_{i,t-1}+\varepsilon_{t,t,}\Lambda_{i}$$ (6)

$$\sum_{t}={DD}^{T}+C_{1}(\varepsilon_{t-1}\varepsilon_{t-1}^{T})C_{1}^{T}+E_{1}\Sigma_{t-1}E_{1}^{T}$$ (7)

Where *R*_*i*,*t*_ is an *n x 1* parameter of daily returns at time *t* for our selected markets and *ε*_*i*,*t*_/*I*_*t*−1_~*N*(0, *K*_*t*_). The *n x 1* is the parameter of random errors, *ε*_*i*,*t*_ is the origination for individual stock market at time t with its corresponding *n x n* conditional variance-covariance matrix, *K*_*t*_. The stock market data accessible at time *t − 1* is denoted by the data set *I*_*t*−1_. The *n × 1* parameter, *α*, signify the long-term drift factors.

The features *α*_*ij*_ of the matrix A can specify the processes of the impact of the spillovers across periods. In the BEKK framework, *d*_*ij*_ are components of an *n × n* symmetric matrix of constants *D*, the elements *c*_*ij*_ of the symmetric *n × n* matrix *C* measure the level volatility spillovers from the stock market *i* to *j*, and the features *e*_*ij*_ of the symmetric *n × n* matrix *E* signify the insistence in conditional volatility between the stock market *i* and stock market *j*.

### 4.3 VAR model

The VAR model is developed by Sims (1980) [68]. The VAR model is appropriate to detention the volatility transmission across the markets. So, the conventional VAR model is implemented to conduct a dynamic analysis of the adjustment of the volatilities to shocks arising from different stock markets through variance decomposition examines. We have applied a V.A.R. model if cointegration does not exist. Contrary to, if cointegration exists at *Y*_*t*_, we have applied modelled over the vector error correction model (V.E.C.M.).

The mathematical demonstration of m th order VAR is:

$$Y_{t}=c+\sum_{s=1}^{m}\varphi_{s}Y_{t-s}+\epsilon_{t}$$ (8)

Where *Y*_*t*_ represents a (7×1) column vector of stock market conditional volatility on behalf of the 7 markets under respect, *c* symbolizes a (7×1) vector of constants, *φ*_*s*_ are (7×7) matrix of autoregressive coefficients, *m* is the lag length and the *ϵ*_*t*_ is a vector of innovations that perhaps contemporaneously is correlated but that are consecutively uncorrelated with their particular lagged values and uncorrelated with all of the right-hand side variables.

The moving average receipts the following form:

$$Y_{t}=c+\sum_{s=0}^{k}\delta_{s}\epsilon_{t-s}$$ (9)

Where *Y*_*t*_ represents a linear combination of current and earlier of one step forward forecast error or innovations. It explains the coefficient *δ*_*s*_ that can be interpreted as the reaction of one market returns to one standard error shock of any of the markets under analysis *s* periods back. While, in Eq (8), the *ϵ*_*t*_’s are similarly perhaps contemporaneously being correlated but these are consecutively uncorrelated with their individual lagged values and uncorrelated with all the earlier *Y*_*s*_. Cholesky decomposition is suggested by Sims (1980). This investigation practices the conventional Cholesky decomposition assessment standard to decompose the error components. To execute the short-run dynamic investigation, the GARCH-M assessment is used to be in the *Y*_*t*_ vector.

## 5. Results and discussion

### 5.1 Causality investigation

Since the global financial markets are powerfully integrated due to the progress of technology in this age of globalization, it is quite possible that small and emerging stock markets can also be influenced significantly by mature markets. In order to check the short-run causal link, this study estimates the pairwise granger causality test for the stock market examination. To serve this end, data were turned into stationary series using return series. Furthermore, for the Granger causality that would allow us to research the relationships that are established between variables, it is imperative to choose the correct number of lags. Additionally, if the number of lags is appropriate, there is no autocorrelation left in the residuals. So, the suitability of the number of lags is set with the LM test. The results of the LM test recommend that a lag length of six is suitable for the granger causality test in the crisis, US-China EPU, and COVID-19 periods, another side lag length of three is suitable for the granger causality test in the full, pre-crisis and post-crisis periods.

The results are presented in Table 5. They show that there are noteworthy differences in the causality relationship across the different time periods. The outcomes suggest that at a 1% level of significance US is leading all sample emerging stock markets during the all periods, except for the Bangladesh market (Table 5). India market is found to have bidirectional causality with China and Korean stock market in crisis and COVID-19 periods but bidirectional causality found with Philippine only in post crisis period [31]. Malaysia market is found to have bidirectional causality with US and Philippine market in full period. It is remarkable results that during crisis and COVID-19 periods, few markets have shown bidirectional causality and maximum markets are found to have unidirectional causality with other emerging markets [11]. From those remarks, we imply that absence of interdependence between these markets may be due to different risks and returns that exists in these markets. It can be shown from the Granger causality test that no stock market is performing a very leading role in those emerging markets.

**Table 5:** Pairwise granger causality of the stock markets.

	Full Period		Pre-crisis		Financial Crisis		Post-crisis		US-China EPU		COVID-19	
	*F*-Stat	*p*-Value	*F*-Stat	*p*-Value	*F*-Stat	*p*-Value	*F*-Stat	*p*-Value	*F*-Stat	*p*-Value	*F*-Stat	*p*-Value
BD → US	1.870	0.132	1.151	0.327	0.867	0.519	0.697	0.554	0.611	0.722	0.712	0.593
US → BD	0.595	0.618	**2.707**	0.044	0.599	0.731	**2.873**	0.035	1.657	0.129	0.637	0.791
CN → US	0.617	0.608	1.127	0.337	1.026	0.406	2.227	0.043	0.353	0.908	2.004	0.419
US → CN	**32.162**	0.000	0.468	0.704	**8.921**	0.000	**11.629**	0.000	**10.489**	0.000	**13.364**	0.000
IN → US	1.341	0.259	0.279	0.840	0.663	0.679	1.229	0.298	0.453	0.843	0.518	0.742
US → IN	**84.793**	0.000	**12.067**	0.000	**18.822**	0.000	**35.770**	0.000	**4.482**	0.002	**20.173**	0.000
MY → US	**4.954**	0.002	0.744	0.526	**3.133**	0.005	1.795	0.146	1.156	0.328	2.949	0.185
US → MY	**178.529**	0.000	**32.889**	0.000	**44.547**	0.000	**50.012**	0.000	**5.237**	0.000	**29.168**	0.000
PH → US	2.001	0.112	0.783	0.504	2.073	0.054	2.565	0.053	**2.148**	0.046	**3.005**	0.048
US → PH	**316.925**	0.000	**32.807**	0.000	**94.299**	0.000	**67.381**	0.000	**10.239**	0.000	**75.969**	0.000
KR → US	**8.394**	0.002	**3.277**	0.020	1.565	0.154	**4.526**	0.004	0.607	0.725	2.604	0.103
US → KR	**266.677**	0.000	**60.888**	0.000	**50.709**	0.000	**91.148**	0.000	**14.723**	0.000	**41.906**	0.000
BD → CN	0.058	0.982	0.656	0.579	0.441	0.852	0.273	0.845	0.899	0.495	0.317	0.735
CN → BD	0.576	0.631	1.147	0.329	0.152	0.989	0.771	0.510	1.057	0.387	0.271	0.816
BD → IN	1.428	0.232	0.274	0.844	0.925	0.475	0.972	0.405	0.482	0.822	0.894	0.183
IN → BD	2.244	0.081	**3.914**	0.008	0.539	0.779	2.040	0.107	1.658	0.129	1.027	0.391
BD → MY	1.281	0.279	0.494	0.687	1.084	0.369	0.371	0.774	0.561	0.761	0.923	0.495
MY → BD	1.019	0.383	0.537	0.657	0.921	0.479	1.038	0.375	1.015	0.414	0.805	0.501
BD → PH	1.295	0.274	1.151	0.327	1.475	0.183	0.229	0.876	0.759	0.602	0.852	0.152
PH → BD	1.577	0.193	1.717	0.162	0.997	0.425	2.571	0.053	1.899	0.078	0.839	0.281
BD → KR	0.671	0.570	0.568	0.636	0.308	0.933	1.354	0.255	0.662	0.681	0.416	0.943
KR → BD	0.881	0.448	1.805	0.144	0.244	0.962	**4.503**	0.004	1.045	0.395	0.947	0.829
CN → IN	**2.845**	0.036	0.678	0.565	**3.685**	0.001	1.009	0.388	1.261	0.273	**3.027**	0.041
IN → CN	**7.888**	0.015	0.831	0.477	**2.771**	0.011	2.346	0.071	0.799	0.571	**4.319**	0.038
CN → MY	1.894	0.128	2.065	0.103	1.523	0.167	1.852	0.136	1.054	0.389	2.175	0.125
MY → CN	0.700	0.552	**2.939**	0.032	1.839	0.088	1.105	0.346	1.737	0.109	2.809	0.075
CN → PH	**5.531**	0.001	**2.829**	0.037	**2.160**	0.044	0.371	0.774	1.441	0.196	**3.719**	0.035
PH → CN	0.530	0.661	2.403	0.066	0.454	0.842	1.279	0.280	0.637	0.701	0.813	0.724
CN → KR	1.353	0.255	0.164	0.921	1.334	0.239	0.074	0.974	1.932	0.073	**2.973**	0.039
KR → CN	0.345	0.793	0.242	0.867	0.444	0.849	2.202	0.086	1.114	0.352	1.729	0.372
IN → MY	**25.179**	0.001	2.556	0.054	**10.292**	0.000	**4.520**	0.004	2.096	0.052	2.173	0.194
MY → IN	0.274	0.844	0.998	0.392	1.316	0.247	1.785	0.148	1.362	0.227	2.001	0.397
IN → PH	**68.749**	0.000	**3.591**	0.013	**20.877**	0.000	**19.853**	0.000	**3.155**	0.005	**17.004**	0.028
PH → IN	2.259	0.079	0.378	0.769	1.072	0.377	**5.413**	0.006	0.488	0.818	2.505	0.204
IN → KR	**28.091**	0.000	**6.013**	0.005	**8.747**	0.000	**9.268**	0.000	1.047	0.394	**5.927**	0.006
KR → IN	**6.727**	0.010	0.937	0.422	**2.265**	0.035	**5.288**	0.003	**2.204**	0.041	**3.814**	0.021
MY → PH	**23.116**	0.000	2.471	0.060	**9.319**	0.000	2.412	0.065	**4.669**	0.001	**7.173**	0.008
PH → MY	**2.822**	0.037	0.773	0.509	1.968	0.067	1.992	0.113	0.465	0.834	**2.909**	0.048
MY → KR	2.522	0.056	0.053	0.984	1.616	0.139	0.997	0.393	**3.041**	0.006	2.104	0.291
KR → MY	**9.064**	0.000	1.688	0.168	**3.789**	0.001	2.202	0.086	1.631	0.136	2.826	0.089
PH → KR	**4.377**	0.004	0.976	0.403	1.985	0.065	1.109	0.344	1.808	0.095	2.831	0.073
KR → PH	**27.610**	0.000	1.711	0.163	**10.089**	0.000	**2.632**	0.049	1.742	0.109	**4.573**	0.037

**Note:** Values of t-statistics that are statistically significant at the 5% level are presented in bold face.

### 5.2 Estimates of the GARCH model and volatility spillover

In this section, we present the results of the GARCH-M model. Table 6 presents the results of the market volatility spillover effects from US market news towards the Asian emerging markets. In the Bangladesh market, we find that abnormal persistence of stock market returns volatility in the market for the full period, post-crisis, and COVID-19 periods. The volatility of stock returns is not affected by its own previous returns and more impact of previous own news but US market news does not affect Bangladeshi markets. Further results indicate that without the pre-crisis period the US market news cannot affect the Chinese market. The volatility of stock market returns is affected by its individual earlier returns and the pre-crisis period of Chinese market return has more impact on previous news. The Chinese markets negatively respond to their domestic economic policy uncertainty in the trade war shocks [44]. Moreover, the negative responses of the Chinese markets to the COVID-19 pandemic that is not required a long time to rebalance the markets. Indian market results show that the US market news affects this market, even in full period and crisis period; the US market significantly affects the Indian stock market [46]. The GARCH coefficient indicates that the volatility of stock returns is affected by its own previous returns. During the post-crisis period, Indian markets return have less impact on previous news, but the COVID-19 pandemic affects more this market.

**Table 6:** Asian emerging stock markets- application of GARCH-M model.

		Periods	C(ɷ)	*λ*_1_	*λ*_2_	*α*_0_	*α*_1_	*β*_1_	*α*_1_+*β*_1_	AIC	SIC	Log-Likelihood
**Bangladesh**	**BD**	Full Period	0.0007^c^	0.0645	-0.7574	6.83E-06^a^	0.3621^a^	0.6955^a^	1.0576	-6.1640	-6.1543	12059
		Pre-Crisis	-0.0012^c^	0.2026^b^	-0.4875	2.52E-06^a^	0.1905^a^	0.7986^a^	0.9891	-6.6568	-6.6330	4339
		Crisis	0.0035^b^	-0.0142	-2.6507	6.11E-05^a^	0.3349^a^	0.4963^a^	0.8312	-5.5472	-5.5234	3620
		Post-Crisis	0.0005	0.0106	-1.0965	4.94E-07^b^	0.1726^a^	0.8437^a^	1.0163	-6.6591	-6.6353	4347
		US-China EPU	-0.0022^b^	0.3430^b^	-2.0004	4.89E-06^a^	0.2447^a^	0.6733^a^	0.9180	-7.1716	-7.1340	2637
		COVID-19	0.0018^c^	0.0372	-1.6105	5.13E-05^a^	0.0647^a^	0.7865^a^	0.8512	-6.3471	-6.7231	1625
**China**	**CN**	Full Period	-0.0004	0.0810	-2.2670^b^	2.37E-06^a^	0.0705^a^	0.9218^a^	0.9923	-5.6827	-5.6731	11118
		Pre-Crisis	-0.0023	0.2602^b^	-3.6834	7.71E-06^a^	0.1090^a^	0.8548^a^	0.9638	-5.8767	-5.8528	3831
		Crisis	-0.0020	0.1421	-0.7327^b^	2.17E-06^a^	0.0430^a^	0.9501^a^	0.9931	-5.2283	-5.2044	3412
		Post-Crisis	0.0008	0.0442	-16.716^c^	1.32E-06^a^	0.0561^a^	0.9370^a^	0.9931	-5.9532	-5.9294	3887
		US-China EPU	0.0003	0.1433^a^	-1.0334	5.32E-07^a^	0.0598^a^	0.9395^a^	0.9993	-6.5436	-6.5060	2407
		COVID-19	-0.0031	0.1035^b^	-2.6311^b^	6.14E-05^a^	0.0735^a^	0.9024^a^	0.9759	-5.3261	-5.1341	2401
**India**	**IN**	Full Period	-0.0003	0.1562^b^	-3.9198^a^	3.60E-07^a^	0.1041^a^	0.8778^a^	0.9891	-6.0336	-6.0239	11804
		Pre-Crisis	0.0022^c^	0.0297	-8.7788^b^	1.28E-05^a^	0.1675^a^	0.7474^a^	0.9149	-6.1011	-6.0773	3977
		Crisis	-0.0003	0.1051^b^	-1.9480^a^	3.74E-06^a^	0.1174^a^	0.8797^a^	0.9971	-5.4826	-5.4588	3577
		Post-Crisis	-0.0048^b^	0.6523^a^	-7.0741^c^	3.44E-06^a^	0.0418^a^	0.9170^a^	0.9588	-6.5540	-6.5302	4279
		US-China EPU	0.0007	0.0153	-2.1511^a^	2.48E-06^a^	0.0854^a^	0.8724^a^	0.9578	-7.0463	-7.0087	2591
		COVID-19	-0.0014	0.4308^a^	-1.8531^a^	3.37E-06^a^	0.1021^a^	0.8801^a^	0.9822	-6.9015	-6.0372	2815
**Malaysia**	**MY**	Full Period	-0.0006^b^	0.2119^a^	-1.7066^b^	1.16E-06^a^	0.1137^a^	0.8674^a^	0.9811	-7.2765	-7.2669	14235
		Pre-Crisis	-0.0009	0.2490^c^	-2.2887	1.35E-06^a^	0.0912^a^	0.8813^a^	0.9725	-7.2553	-7.2314	4729
		Crisis	7.57E-5	0.1411	-1.9517^b^	1.66E-06^a^	0.1395^a^	0.8509^a^	0.9904	-6.8317	-6.8079	4456
		Post-Crisis	-0.0009	0.2112	2.4117^c^	2.28E-06^a^	0.1251^a^	0.7946^a^	0.9197	-7.7488	-7.7250	5058
		US-China EPU	-0.0003	0.0596	1.0361^b^	4.23E-07^c^	0.1186^a^	0.8842^a^	1.0028	-7.6476	-7.6100	2812
		COVID-19	-0.0012	0.3018^b^	-1.0815^b^	2.73E-06^a^	0.1403^a^	0.7981^a^	0.9384	-6.0419	-6.1541	2456
**Philippine**	**PH**	Full Period	-0.0007	0.1778^b^	-2.6309^b^	7.09E-06^a^	0.1383^a^	0.8170^a^	0.9553	-6.1817	-6.1721	12094
		Pre-Crisis	-0.0010	0.2424	-8.2703^a^	1.2E-05^a^	0.1293^a^	0.7778^a^	0.9071	-6.2078	-6.1839	4047
		Crisis	-0.0011	0.2040^c^	-1.4437^c^	1.28E-05^a^	0.1728^a^	0.7718^a^	0.9446	-5.8460	-5.8222	3814
		Post-Crisis	-0.0002	0.1064	1.7914	5.58E-06^a^	0.1247^a^	0.8241^a^	0.9488	-6.4935	-6.4697	4239
		US-China EPU	-0.0001	0.0589^c^	-1.0121^b^	2.33E-06^c^	0.0608^a^	0.9126^a^	0.9734	-6.5062	-6.4686	2393
		COVID-19	0.0104	0.1081	-2.5138^c^	3.27E-05^a^	0.1621^a^	0.8013^a^	0.9634	-6.4061	-6.7293	2158
**Korea**	**KR**	Full Period	-0.0007	0.1648^a^	-3.0557^b^	1.44E-06^a^	0.0891^a^	0.9046^a^	0.9937	-6.1251	-6.1155	11983
		Pre-Crisis	0.0021^c^	-0.0574	-2.6192	2.49E-06^b^	0.0727^a^	0.9160^a^	0.9887	-5.7564	-5.7326	3753
		Crisis	-0.0003	0.1590^c^	-3.6072^c^	4.06E-06^a^	0.1184^a^	0.8712^a^	0.9896	-5.6860	-5.6622	3710
		Post-Crisis	-0.0032^b^	0.4692^b^	1.0872	3.38E-06^a^	0.0756^a^	0.8657^a^	0.9413	-6.9508	-6.9271	4537
		US-China EPU	0.0044^a^	-0.5346^a^	-2.1916^a^	9.62E-08^a^	0.0197^a^	0.9756^a^	0.9953	-6.9638	-6.9262	2561
		COVID-19	-0.0013^c^	0.3091^c^	-1.7492^c^	5.16E-06^a^	0.1201^a^	0.8532^a^	0.9733	-5.7831	-5.9632	2013

**Note:** The alphabets a, b, and c denote 1%, 5% and 10% level of statistical significance.

In the Malaysian market, outcomes are almost similar to the Chinese and Indian markets. The US market news has more or less effects on the Malaysian stock markets without the pre-crisis period. The volatility of stock market returns is affected by its individual earlier returns and the return has less impact on previous news in the Malaysian stock market and negative responses found by the COVID-19 pandemic. The Philippine market results show that the US market news has effects on this market, the only exception is discovered in the post-crisis market period. The volatility of stock returns has been affected by its own previous returns and market return which has less impact on previous news in the Philippine stock market. South Korean market results indicate that without pre-crisis and post-crisis periods the US market news affects the South Korean stock market. The GARCH coefficient and ARCH coefficient are indicating that the volatility of stock returns is affected by its own previous returns and market return has less impact on previous news in the South Korean stock market [49].

In particular, in the stock markets of China, India, and Malaysia from Asian emerging stock markets, many fluctuations are found during the global financial crisis period 2007–2008, US-China EPU 2018–2019, and the COVID-19 pandemic. The Asian emerging markets are affected by their own past shocks. Particularly, these are more focused on Bangladeshi stock market results as compared to the other Asian emerging markets. The US stock market is highly correlated with Asian emerging stock markets in terms of market volatility, especially during the global financial crisis period 2007–2008 and less in the US-China EPU period 2018–2019 and the COVID-19 pandemic. In this investigation, results find that in 2007–2008; the Chinese, Indian, and Malaysian stock markets are hardly affected by US stock markets in terms of volatility spillover. Economic policy uncertainty news at US-China EPU period 2018–2019 results indicate that the Chinese stock markets are very badly affected by US stock markets in terms of volatility spillover [9]. Prior to the COVID-19 pandemic, the Chinese, Malaysian, and South Korean stock markets are slightly affected by the US stock market news. But, most of the markets are negatively influenced by the COVID-19 pandemic.

### 5.3 Dynamic analysis of the conditional volatility

The results for the V.E.C.M are given in the S1 Appendix. The results show that there are long-run causalities among the US and Asian emerging stock markets. The results revealed that coefficients of error correction terms are not statistically significant in case of US stock market. In contrast, it is statistically significant for Asian emerging stock markets. The results do not show a causal relationship between Bangladesh’s stock returns and US stock returns but the cointegration vector system is statistically significant. The results revealed that US stock market lead other markets suggesting that any external news is arrives simultaneously get affected by US stock market and then transmitted to other markets.

Tables 7 and 8 show the results of the variance decompositions that are analyzed using the Cholesky factorization-based variance decompositions, where the stock markets are ordered as exhibited. Both tables expose the order dependence of the variance decompositions. The result shows that Bangladesh’s market return is not affected by other countries’ markets. The volatility of the Bangladesh market has been described by the shocks of its individual market but the Indian market shock starts to rise notably the volatility in the Bangladesh market and their outcomes are nearly similar to this outcome [57]. As the Bangladesh market is basically relatively small compare to other Asian emerging markets, this stock market is still not open like the other five emerging stock markets [69]. A maximum of 8% of the variation in China market return spillover can be described through the US market. From Asian emerging region markets, Malaysia has simply the effect on the China stock market. US market shock starts to notably rise the volatility in the China stock market. The China market has a strong influence on its border country when the China market increases because China is a vital trading partner and strategic financial center indications to exert a noteworthy impact on the Asian emerging markets [44]. Additionally, when the Chinese domestic market performs better, it will show a comparably positive effect on the global economy. India’s market is major affected by China and Malaysia stock markets. China stock market shock starts to raise notably the return in India stock market from the 1 period on. On the other side, from US stock market has also had a significant impact on India’s stock market return. The US and China stock markets have lagged with noteworthy impacts on India’s stock market volatilities.

**Table 7:** Asian emerging markets return spillovers.

Lag	Bangladesh	China	India	Malaysia	Philippine	Korea	US
**A. Percentage of conditional volatility of Bangladesh stock returns explained by returns of**							
1	100.00	0.00	0.00	0.00	0.00	0.00	0.00
5	99.64	0.03	0.13	0.04	0.11	0.01	0.04
10	99.64	0.03	0.13	0.04	0.11	0.01	0.04
20	99.64	0.03	0.13	0.04	0.11	0.01	0.04
40	99.64	0.03	0.13	0.04	0.11	0.01	0.04
50	99.64	0.03	0.13	0.04	0.11	0.01	0.04
**B. Percentage of conditional volatility of China stock returns explained by returns of**							
1	0.00	100.00	0.00	0.00	0.00	0.00	0.00
5	0.00	94.01	0.04	0.56	0.06	0.02	5.31
10	0.00	92.50	0.06	1.01	0.06	0.02	6.35
20	0.00	89.40	0.06	2.11	0.06	0.02	8.35
40	0.00	89.05	0.06	2.06	0.06	0.02	8.75
50	0.00	89.55	0.06	2.01	0.06	0.02	8.30
**C. Percentage of conditional volatility of India stock returns explained by returns of**							
1	0.00	2.87	96.11	0.00	0.00	0.00	1.03
5	0.00	2.78	89.04	1.32	0.14	0.26	6.46
10	0.00	2.78	89.04	1.32	0.14	0.26	6.46
20	0.00	2.82	89.00	1.42	0.14	0.26	6.36
40	0.00	2.82	89.00	1.42	0.14	0.26	6.36
50	0.02	2.82	88.00	1.40	0.14	0.26	7.36
**D. Percentage of conditional volatility of Malaysia stock returns explained by returns of**							
1	0.00	3.18	6.95	89.84	0.00	0.00	0.03
5	0.00	2.89	8.76	77.91	0.22	0.10	10.12
10	0.00	2.89	8.76	77.91	0.20	0.12	10.12
20	0.00	2.89	8.76	77.91	0.18	0.14	10.12
40	0.00	2.89	8.76	77.91	0.16	0.16	10.12
50	0.01	2.89	8.76	77.91	0.16	0.15	10.12
**E. Percentage of conditional volatility of the Philippine stock returns explained by returns of**							
1	0.00	1.30	2.77	6.00	89.92	0.01	0.00
5	0.00	1.56	7.69	5.63	70.53	0.47	14.12
10	0.00	1.56	7.69	5.63	70.53	0.47	14.13
20	0.02	1.56	7.69	5.63	70.53	0.45	14.13
40	0.02	1.56	7.69	5.63	70.53	0.45	14.13
50	0.02	1.56	7.69	5.63	70.53	0.45	14.13
**F. Percentage of conditional volatility of the Korea stock returns explained by returns of**							
1	0.00	3.27	10.43	6.22	1.10	78.98	0.00
5	0.00	2.86	10.74	5.32	1.11	66.50	13.48
10	0.00	2.86	10.74	5.32	1.11	66.50	13.48
20	0.00	2.86	10.74	5.32	1.11	66.50	13.48
40	0.01	2.85	10.74	5.32	1.11	66.50	13.48
50	0.01	2.85	10.74	5.32	1.11	66.50	13.48
**G. Percentage of conditional volatility of the US stock returns explained by returns of**							
1	0.00	0.78	7.21	0.38	0.05	1.78	89.79
5	0.00	0.82	7.09	0.74	0.15	2.02	89.19
10	0.00	0.82	7.09	0.74	0.15	2.02	89.19
20	0.00	0.92	7.09	0.74	0.05	2.02	89.19
40	0.00	0.92	7.09	0.74	0.05	2.02	89.19
50	0.02	0.90	7.09	0.74	0.05	2.02	89.19

**Data source:** The sample from January 2, 2002 to December 30, 2020. The number of the lags in the VAR is 2. The (i, j) th value is the estimated contribution to the variance of the 8-week-ahead stock return forecast error of country i coming from innovations to the stock return of country j. The Choleski decomposition is in the following order: Bangladesh, China, India, Malaysia, Philippine, South Korea, and United States. Using other numbers of lags or orders of decomposition yields similar results.

**Table 8:** Asian emerging markets volatility spillovers.

Lag	Bangladesh	China	India	Malaysia	Philippine	Korea	US
**A. Percentage of conditional volatility of Bangladesh stock returns explained by conditional volatilities of returns of**							
1	100.00	0.00	0.00	0.00	0.00	0.00	0.00
5	99.89	0.00	0.02	0.07	0.00	0.02	0.00
10	99.48	0.00	0.17	0.23	0.03	0.08	0.01
20	98.41	0.01	0.89	0.40	0.05	0.21	0.02
40	96.44	0.03	2.68	0.41	0.09	0.34	0.03
50	95.73	0.03	3.29	0.40	0.16	0.35	0.04
**B. Percentage of conditional volatility of China stock returns explained by conditional volatilities of returns of**							
1	0.00	100.00	0.00	0.00	0.00	0.00	0.00
5	0.00	91.30	0.43	1.10	0.25	0.22	6.70
10	0.00	84.65	0.28	2.41	0.20	1.19	11.27
20	0.00	77.90	1.19	5.72	0.14	1.27	13.78
40	0.02	74.14	1.55	4.77	0.23	2.31	16.00
50	0.02	74.50	2.50	4.74	0.42	2.40	15.44
**C. Percentage of conditional volatility of India stock returns explained by conditional volatilities of returns of**							
1	0.07	2.87	97.06	0.00	0.00	0.00	0.00
5	0.00	4.84	91.71	0.22	0.45	0.19	2.59
10	0.00	5.97	83.07	1.09	1.80	0.25	7.83
20	0.00	7.71	71.53	2.57	4.90	0.45	12.83
40	0.02	10.26	60.09	3.60	8.58	0.75	16.70
50	0.09	11.18	60.32	3.72	9.28	0.75	14.66
**D. Percentage of conditional volatility of Malaysia stock returns explained by conditional volatilities of returns of**							
1	0.00	0.83	3.80	95.34	0.03	0.00	0.00
5	0.00	2.46	4.92	90.39	0.43	0.15	1.65
10	0.00	4.42	4.45	84.35	0.64	0.24	5.89
20	0.00	8.23	4.06	75.63	0.82	0.34	10.91
40	0.00	13.45	4.28	69.27	0.79	0.41	11.79
50	0.00	15.12	4.41	67.47	0.81	0.44	11.75
**E. Percentage of conditional volatility of the Philippine stock returns explained by conditional volatilities of returns of**							
1	0.00	3.64	6.06	3.95	86.35	0.00	0.00
5	0.00	4.08	5.29	3.48	84.58	0.50	2.08
10	0.00	4.10	4.85	3.71	84.62	0.58	2.14
20	0.01	7.99	4.38	3.91	80.91	0.76	2.04
40	0.00	8.85	4.19	3.92	76.16	0.94	5.96
50	0.03	8.82	4.08	3.91	75.17	1.04	6.95
**F. Percentage of conditional volatility of the Korea stock returns explained by conditional volatilities of returns**							
1	0.00	0.14	5.34	0.33	0.60	93.58	0.00
5	0.00	1.46	4.58	0.71	1.85	86.45	4.94
10	0.00	3.27	3.97	1.34	4.20	80.80	6.43
20	0.00	4.37	3.16	2.46	8.11	72.82	9.09
40	0.03	6.84	2.96	2.65	10.49	67.19	9.84
50	0.02	9.69	3.03	2.63	10.60	66.47	7.56
**G. Percentage of conditional volatility of the US stock returns explained by conditional volatilities of returns of**							
1	0.00	1.10	8.57	0.03	0.49	1.78	88.04
5	0.00	3.16	5.53	0.47	0.42	2.38	88.05
10	0.00	4.65	5.00	1.91	1.01	2.44	84.99
20	0.00	6.98	4.61	3.41	1.91	2.43	80.66
40	0.00	9.80	4.90	3.60	2.12	2.37	77.21
50	0.06	10.62	5.05	3.56	2.10	2.35	76.27

**Data source:** The sample from January 2, 2002 to December 30, 2020. The number of the lags in the VAR is 2. The (i, j) th value is the estimated contribution to the variance of the 8-week-ahead stock return volatility forecast error of country i coming from innovations to the stock return volatility of country j. The Choleski decomposition is in the following order: Bangladesh, China, India, Malaysia, Philippine, South Korea, and United States. Using other numbers of lags or orders of decomposition yields similar results.

The US, India, and China stock markets have lagged significant influences on Malaysia stock market return spillover. China and US stock markets shock start to notably increase the volatility in the Malaysia stock market. The China stock market effect describes 15% variability in Malaysia stock market return volatility. Malaysia and India from the Asian emerging stock markets have lagged influence on Philippine stock market return spillover from the 5 periods on. The notable effect from the US market is considered as the large trading partnership with good friendship history. The US and China markets shock start to significantly influence the volatility in the Philippine market. South Korea’s market return is largely affected by India and Malaysia markets in Asian emerging countries but mostly is affected by the US market news. The Philippine and US markets have lagged significant impacts on South Korean market volatilities. It is clear that the US market return is not affected by the other stock markets in the Asian emerging stock markets, except the Indian stock market. A maximum of 7% of the variation in the US stock market can be explained by the Indian market. China’s market shock significantly increases the volatility in the US stock market. The majority of the US stock market volatility is explained by its own shocks. Subsequently, the parallel of outcomes is found between the modeling approaches which are actually notable. Even though emerging markets are similarly very volatile, particularly it is driven by bad news from the financial markets, and their massive volatility is spread back to the US stock market.

## 6. Conclusions

In this paper, we investigate the Granger causality and volatility spillover effects among six selected emerging Asian stock markets and the US stock market in various crisis periods. To this end, we apply the Granger causality test, GARCH-M model, and VAR model on daily stock index data from January 2002 to December 2020.

This paper demonstrates that volatility and return spillovers differ over time, during the pre-crisis, crisis, post-crisis, US-China EPU, and COVID-19 periods. The negative shock of the US stock market affects the selected six emerging Asian stock markets, especially in the global financial crisis, US-China EPU, and COVID-19 periods, with the only exception found in Bangladesh [7], which reflects its segmentation and herby its possible benefits of diversification when combined in the same portfolio with the other stock markets. The outcomes show that while the sample markets are segmented before the global financial crisis and US-China EPU periods, they have become highly correlated during the global financial crisis and COVID-19 periods and comparatively less correlated US-China EPU period. The results indicate that a superior level of risk presents in emerging markets during the crisis period than in the EPU period and post-crisis period [70]. This indicates that Asian markets are closely connected to the more developed US stock market. Bangladesh, India, and China markets are not deeply affected by US stock markets in terms of return spillover. However, only the global financial crisis period return spillovers in the Asian emerging territory touches the peak level. The negative responses of the Chinese markets to the economic policy uncertainty from the US and the COVID-19 pandemic are not required for a long time to rebalance the markets.

The stock market indices of emerging countries are characterized by ups and downs periods, which has been seen while working on six emerging Asian markets, particularly in China and Bangladesh markets where we notice high volatility. The market shows an extraordinary return rate which is sometimes 100% per month and has regularly remained at the level of 40% per month which is the maximum among major national stock markets [71]. Emerging Asian stock markets are shaped by global events and this has been outlined by the outcomes from the granger causality test, GARCH-M, and VAR models that investigated the level of integration in the global financial crisis period and US-China EPU period with respect to the US stock market. Moreover, considering the fact that US-China economic policy uncertainty has appeared with adverse policies against China, on the contrary, the US is not encouragingly progressing further relations with the world’s second-largest Chinese economy.

The findings have relevant implications for policymakers in the context of the emerging stock markets of Asian countries. Because, there are no developed economies in the world that can deal with the growth of emerging Asian countries, recently researchers and economists express their opinion that the next era is for Asian economics. Specifically, international portfolio managers and investors can now better understand how the volatility linkage between markets connected over time; our analysis might provide them an advantage in predicting the behavior of this market by taking the other market evidence. It is observed that the repeated government involvement can also strengthen the remarked government guarantees by market participants, which in turn creates another channel to treat extreme risk-taking by market participants. Zhu (2016) points out the whole account of how government assurances have managed extensive risk-taking behavior across the individual countries [72]. Shortly, the share market will appear a lot more appealing to individuals, if the emerging Asian country’s stock markets restructurings their rules and policy as market-oriented, and the investors both domestic and international will provide the possibility for great investment chances and improved use of resources.

## Supporting information

S1 Appendix (DOCX)

S1 Data (XLSX)
