# Supplementary material for: Emerging stock market reactions to shocks during various crisis periods
Source: PLoS One. 2022 Sep 13;17(9):e0272450. doi: 10.1371/journal.pone.0272450 (PMC9469992; doi:10.1371/journal.pone.0272450)
Supplement: S1 Appendix — (DOCX) [file pone.0272450.s001.docx]

Appendix A

1. Results of Vector Error Correction Model (V.E.C.M.)

| Error correction: | Bangladesh | China | India | Malaysia | Philippine | Korea | US |
| --- | --- | --- | --- | --- | --- | --- | --- |
| ECT | 0.118881  [1.40266] | 0.000841  [2.07931]* | -0.529109  [-3.01562]* | -0.310621  [-1.70271]* | -0.739023  [-3.91071]* | 0.000512  [2.00137]* | 0.000005  [1.53092] |
| Bangladesh(-1) | 0.101079  [2.85361]* | 0.005173  [0.20182]* | -0.025291  [-1.70414]* | 0.000106  [0.03161] | 0.000294  [0.02005] | 0.000106  [0.00517] | -0.004107  [-1.08503] |
| Bangladesh(-2) | -0.182460  [-3.78251] | -0.039104  [-1.71503] | -0.079105  [-1.04281] | -0.001951  [-1.95035] | -0.006081  [-1.61104] | -0.051061  [-0.07016] | -0.003196  [0.91051] |
| China (-1) | 0.021804  [0.31305] | -0.050219  [-2.49021]* | -0.049074  [-2.81411]* | -0.041905  [-1.90503] | -0.193105  [-1.08033] | -0.004917  [-2.00613]* | -0.008151  [-3.79014] |
| China (-2) | -0.170452  [-2.78253] | -0.040813  [-1.51041] | -0.039012  [-1.74282] | -0.021652  [-1.85431] | -0.092023  [-1.71201] | -0.039068  [-0.48013] | 0.001143  [0.65053] |
| India (-1) | 0.011301  [0.42311] | -0.060212  [-2.14021]* | -0.036103  [-2.61422]* | -0.051816  [-1.71004]* | -0.152128  [-1.05132] | -0.005013  [-2.00312] | -0.004245  [-2.81192] |
| India (-2) | -0.210061  [-2.52021] | -0.010439  [-1.15721] | -0.070592  [-1.08421] | -0.005193  [-1.03955] | -0.096024  [-1.53127] | -0.030611  [-0.06022] | -0.009315  [0.70511] |
| Malaysia (-1) | 0.190107  [2.15386] | 0.007513  [0.18202]* | -0.022953  [-1.64104]* | 0.000601  [0.01613]* | 0.000946  [0.01529] | 0.000614  [0.00751] | -0.002181  [-1.05082] |
| Malaysia (-2) | -0.145207  [-2.82573] | -0.014082  [-1.10452] | -0.042901  [-1.64228] | -0.032165  [-1.54383] | -0.041823  [-1.20173] | -0.070683  [-0.23801] | 0.008431  [0.53650] |
| Philippine (-1) | 0.149183  [1.26407] | 0.000384  [2.19602] | -0.710355  [-2.15051] | -0.623101  [-1.17127] | -0.836191  [-3.01491]* | 0.000261  [1.00371] | 0.000029  [1.35207] |
| Philippine (-2) | -0.517047  [-2.82637] | -0.048137  [-1.04516] | -0.013029  [-1.27485] | -0.052164  [-1.54832] | -0.072325  [-1.41237] | -0.063917  [-0.93801] | 0.007314  [0.50562] |
| Korea (-1) | 0.052081  [0.40135] | -0.015029  [-2.90241] | -0.074916  [-2.91216] | -0.075419  [-1.50918] | -0.231085  [-1.09381] | -0.001493  [-2.06117]* | -0.001511  [-3.21904] |
| Korea (-2) | -0.713182  [-3.25205] | -0.041035  [-1.53211] | -0.059771  [-1.05214] | -0.009514  [-1.07391] | -0.086225  [-1.31523] | -0.059923  [-0.07163] | -0.003951  [0.42105] |
| US (-1) | 0.207162  [-3.81291] | 0.001394  [2.31641]* | -0.159207  [-2.15013]* | -0.610724  [-1.91403]* | -0.382193  [-2.04115]* | 0.008119  [1.00249]* | 0.000007  [1.41916] |
| US (-2) | -0.296451  [-3.53237] | -0.013408  [-1.61912] | -0.057239  [-1.48291] | -0.041358  [-1.71739] | -0.022985  [-1.90569] | -0.039194  [-0.71916] | 0.004839  [0.17937] |

Note: ECT represents the lagged error correction term; * indicates 1% level of significance; figures in parentheses are t values; lag length is chosen based on AIC and FPE and found to be 2.
